# The Dopaminergic Cells in the Median Raphe Region Regulate Social Behavior in Male Mice

**DOI:** 10.3390/ijms25084315

**Published:** 2024-04-13

**Authors:** Tiago Chaves, Bibiána Török, Csilla Lea Fazekas, Pedro Correia, Eszter Sipos, Dorottya Várkonyi, Zsuzsanna E. Tóth, Fanni Dóra, Árpád Dobolyi, Dóra Zelena

**Affiliations:** 1 Institute of Physiology, Medical School, Centre for Neuroscience, Szentágothai Research Centre, University of Pécs, H7624 Pécs, Hungary; tiagochaves91@gmail.com (T.C.); torok.bibiana@gmail.com (B.T.); ghalla195@gmail.com (C.L.F.); correiaufpe@gmail.com (P.C.); dorka0720@gmail.com (D.V.); 2 Laboratory of Behavioral and Stress Studies, Institute of Experimental Medicine, H1083 Budapest, Hungary; sipos.eszter@koki.hu; 3 János Szentágothai School of Neurosciences, Semmelweis University, H1085 Budapest, Hungary; 4 Laboratory of Neuroendocrinology and in Situ Hybridization, Department of Anatomy, Histology and Embryology, Semmelweis University, H1094 Budapest, Hungary; ztothztoth@gmail.com; 5 Human Brain Tissue Bank, Laboratory of Neuromorphology, Department of Anatomy, Histology and Embryology, Semmelweis University, H1094 Budapest, Hungary; dora.fanni@med.semmelweis-univ.hu; 6 Laboratory of Molecular and Systems Neurobiology, Department of Physiology and Neurobiology, Eötvös Loránd University, H1117 Budapest, Hungary; dobolyi.arpad@ttk.elte.hu

**Keywords:** dopamine, median raphe region, behavior, DREADD

## Abstract

According to previous studies, the median raphe region (MRR) is known to contribute significantly to social behavior. Besides serotonin, there have also been reports of a small population of dopaminergic neurons in this region. Dopamine is linked to reward and locomotion, but very little is known about its role in the MRR. To address that, we first confirmed the presence of dopaminergic cells in the MRR of mice (immunohistochemistry, RT-PCR), and then also in humans (RT-PCR) using healthy donor samples to prove translational relevance. Next, we used chemogenetic technology in mice containing the Cre enzyme under the promoter of the dopamine transporter. With the help of an adeno-associated virus, designer receptors exclusively activated by designer drugs (DREADDs) were expressed in the dopaminergic cells of the MRR to manipulate their activity. Four weeks later, we performed an extensive behavioral characterization 30 min after the injection of the artificial ligand (Clozapine-N-Oxide). Stimulation of the dopaminergic cells in the MRR decreased social interest without influencing aggression and with an increase in social discrimination. Additionally, inhibition of the same cells increased the friendly social behavior during social interaction test. No behavioral changes were detected in anxiety, memory or locomotion. All in all, dopaminergic cells were present in both the mouse and human samples from the MRR, and the manipulation of the dopaminergic neurons in the MRR elicited a specific social response.

## 1. Introduction

It has long been known that mental illness can affect different behaviors resulting in social dysfunctions [1]. Psychopathologies such as schizophrenia, autism spectrum disorder and depression are all good examples of how social relationships can be severely impacted [2,3,4]. According to current scientific evidence, environmental factors and multiple risk genes are the key features for the development of psychopathologies, causing subtle changes in the brain neurotransmission that ultimately lead to behavioral symptoms and emotional instability. One brain structure that is reportedly involved in many mental disorders is the serotoninergic median raphe nucleus.

The median raphe region (MRR, also known as the superior central nucleus, B5+B8 [5]) is located in the midline of the brainstem, and it is constituted by the median and the paramedian raphe regions [6]. It is also part of the mesolimbic serotonergic pathway that projects to the septum and to the hippocampus [7,8]. This pathway has been the subject of a large number of studies, which demonstrate the involvement of MRR in anxiety [9,10,11,12,13], social behavior [14,15,16,17], depression [18] and in the control of the circadian rhythms [19] by regulating the theta rhythms of the hippocampus [20,21].

Although the MRR is widely known as a serotoninergic nucleus, recent studies have shown that serotoninergic neurons are only a minority of all the neurons in the MRR (8.5% of all MRR neurons) and instead, the majority of MRR neurons are GABAergic (61%) or can be characterized by the presence of glutamatergic transporters (vesicular glutamate transporter 2 (VGluT2) and 3 (VGluT3)) [6,22]. The presence [23,24,25] and production [26,27,28,29] of dopamine (DA) in the MRR has repeatedly been confirmed in rats, and its glial uptake—suggesting functional relevance—was also demonstrated [30]. It was even suggested that the hippocampus might receive DAergic innervation from the MRR [31]. The presence of DA was also found in the MRR of deer mice, Peromyscus maniculatus [32], and its production was also shown in chickens [33]. Nevertheless, most of the studies used tyrosine hydroxylase (TH) immunohistochemistry, which is the rate-limiting enzyme of catecholamine synthesis, including DA, noradrenaline and adrenaline. In rats, a considerable amount of noradrenaline was also detected in the MRR [23,24,25], therefore, confirmation of the dopamine-β-hydroxylase (DBH, the key enzyme for noradrenaline synthesis) negativity of the TH positive cells seemed to be important [34]. Moreover, one study on rats showed no TH immunopositivity at all in the MRR, although the authors focused primarily on the dorsal raphe (DR) [35].

DA is present both in the central and peripheral nervous system, and it has been described as a key player in the regulation of a wide range of behaviors [36]. Imbalances of DA can contribute to the emergence of neurological and psychiatric disorders with disturbances in mood, locomotion and cognitive functions [37,38] such as Parkinson’s disease, schizophrenia, addiction and attention hyperactivity disorders [39,40,41,42]. Despite each condition having a different etiology, most of these disorders are characterized by abnormal social behavior. Moreover, in some cases (especially in Parkinson’s disease and schizophrenia), the major target of presently available therapies is the dopaminergic system [43,44,45].

**Figure 1:**
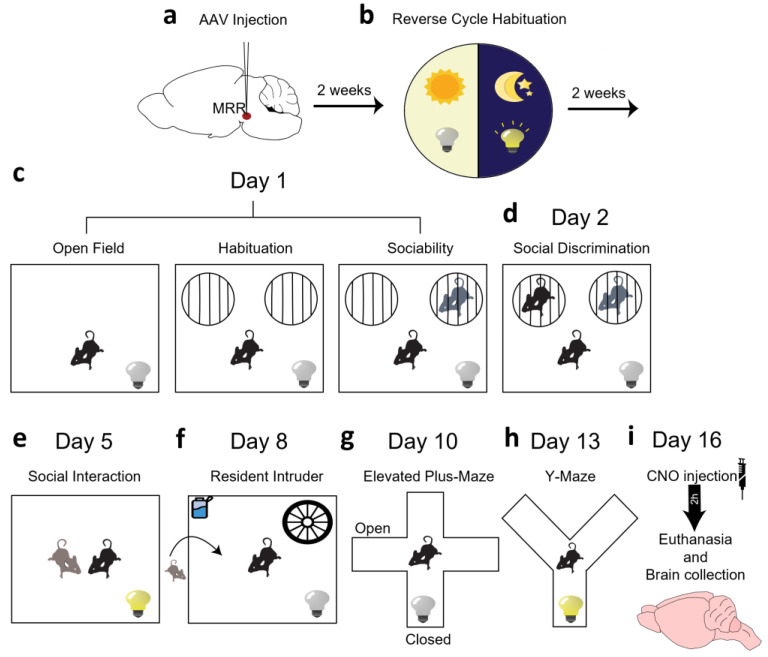
Timeline of the behavioral test battery. Thirty minutes before all experiments (except social discrimination test (SD)) animals were injected intraperitoneally with clozapine-N-oxide (CNO, 1 mg/10 mL/kg diluted in saline). (**a**) Adeno-associated viral (AAV) vectors (20 nL) containing control, stimulatory or inhibitory designer receptor exclusively activated by designer drug (DREADD) sequences were microinjected into the median raphe region (MRR; anteroposterior: −4.1 mm; mediolateral: 0 mm; dorsoventral: −4.6 mm from Bregma) of dopamine transporter-Cre mice. For details see methods section. (**b**) Animals had 14 days to recover after surgery and an additional 14 days to habituate to reversed light–dark cycle. This 28-day incubation time is also enough for DREADD expression. (**c**) On the first test day, 5 min open field (OF), 5 min object habituation and then 5 min sociability tests were conducted. (**d**) On Day 2, an SD test was performed for 5 min to measure social memory. (**e**) On Day 5, social behavior was measured by social interaction test (SIT) for 10 min. (**f**) On Day 8 the resident–intruder test (RIT) was performed for 10 min to investigate aggressive behavior. (**g**) On Day 10, to study anxiety-like behavior, an elevated plus maze (EPM) test was used for 5 min. (**h**) On Day 13, a Y-maze test was conducted for 5 min to measure working memory. (**i**) On the 16th experimental day animals received a final CNO injection, and then 2 h later were euthanized and transcardially perfused. Brain tissue samples were collected for further immunohistochemical analysis.

Our aim was (i) to confirm the presence of dopaminergic cells in the MRR of mice (by immunohistochemistry and RT-PCR) as well as in humans (by RT-PCR); (ii) investigate their role in social behavior using chemogenetics (virally introduced designer receptors exclusively activated by designer drugs (DREADDs) and administration of their synthetic ligand Clozapine-N-oxide (CNO) [46,47]) in dopamine transporter-Cre (DAT-Cre) animals (Figure 1); and (iii) summarize additional information on locomotion, anxiety and memory gained during the behavioral testing, as alterations in these behavior might influence the outcome of social tests.

## 2. Results

### 2.1. Presence of Dopaminergic Cells in the MRR

**Figure 2:**
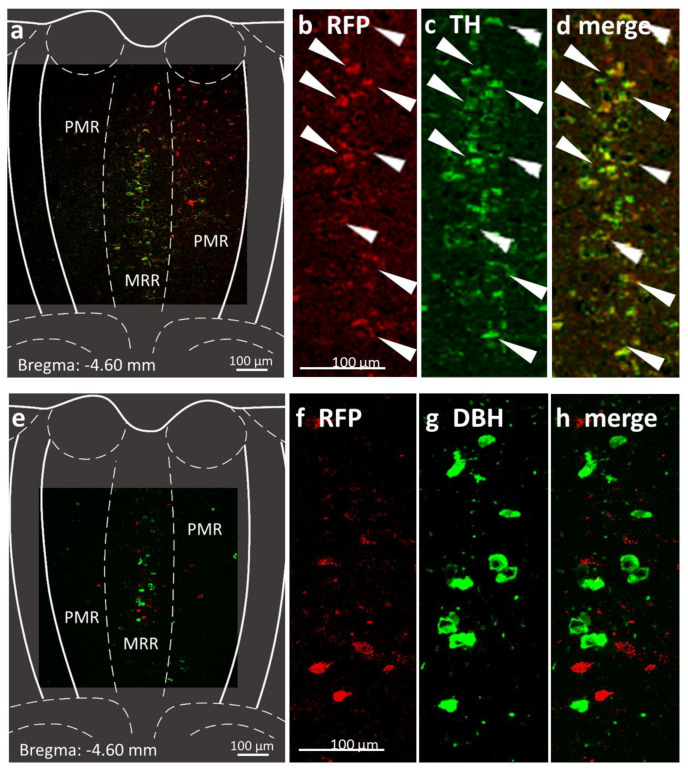

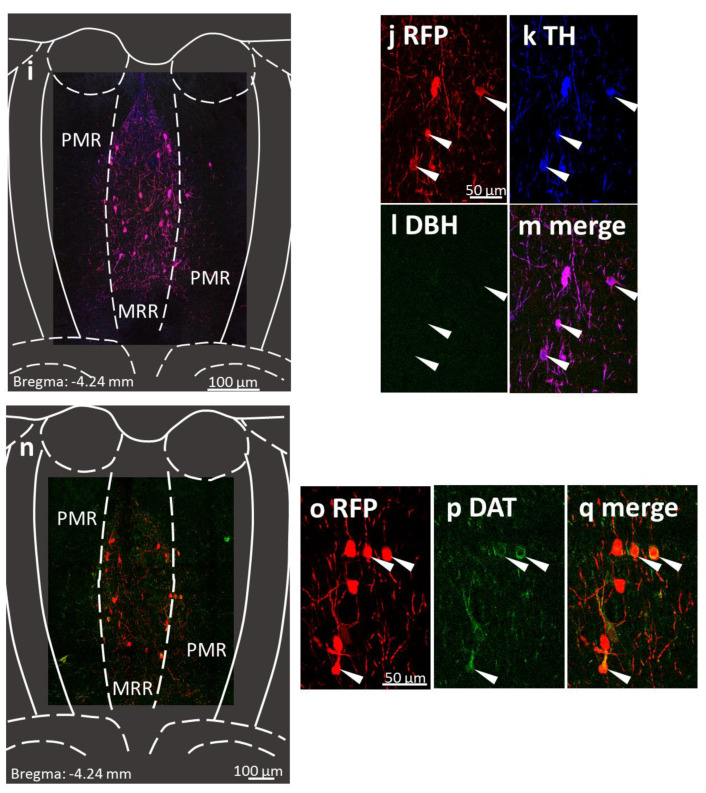
Confirmation of the chemogenetic technique by immunohistochemistry 6 weeks after injection of rAAV8/hSyn-DIO-mCherry (Addgene #50459) into the median raphe region (MRR) of dopamine transporter Cre (DAT-Cre) mice. Subregions (MRR and PMR/paramedian raphe/) are defined based on the Mouse Brain Atlas [49]. Position of the coronal section is indicated in each image, relative to the Bregma. (**a**) Fluorescent micrographs show representative MRR sections with red fluorescent protein (RFP; mCherry, the fluorophore suggesting the presence of the designer receptor exclusively activated by designer drug (DREADD); (red) and tyrosine hydroxylase (TH, rate-limiting enzyme of catecholamine synthesis; green) labelling. (**b**–**d**) Confocal laser scanning images show high co-localization between the labelling of RFP and TH in representative MRR neurons (arrows). (**e**) Fluorescent micrographs show representative MRR sections with RFP (red) and dopamine beta-hydroxylase (DBH, the enzyme which catalyzes the conversion of dopamine to norepinephrine; green) labelling. (**f**–**h**) Confocal laser scanning images show no co-localization between the labelling of RFP and DBH. (**i**–**m**) Confocal laser scanning images of triple immunohistochemical staining of DREADD marker RPF-positive, TH-positive but DBH-negative neurons in the MRR, indicating dopaminergic phenotype. (**n**–**q**) Confocal laser scanning images show co-localization of RFP and DAT.

Double immunohistochemistry of the MRR showed the presence of TH-positive cells, which were co-localized with red fluorescent protein (RFP) aimed to represent the dopaminergic cells in DAT-Cre mice (Figure 2a–d). As described earlier [48], by using an antiserum against TH, we are able to visualize the cells which are involved in catecholamine synthesis, but we cannot discriminate amongst dopaminergic, noradrenergic or adrenergic cells. However, the DBH negativity of the TH-positive cells suggests that DA is the end product. Indeed, the DBH-positive MRR cells of the DAT-Cre mice did not co-localize with the RFP-labelled cells (Figure 2e–h).

In a separate series we conducted triple immunohistochemistry, visualizing RFP, TH and DBH on the same slides of control virus vector injected DAT-Cre animals (Figure 2i–m), which confirmed the previously observed TH positivity and DBH negativity of the RFP-labelled cells.

Using double fluorescent immunohistochemistry against RFP and DAT, we further supported the presence of DAT in these neurons (Figure 2n–q).

These results not only confirm the presence of TH-positive and DBH-negative dopaminergic cells in the MRR of mice but support that these cells (subsequently called DAT-MRR cells) can be effectively labelled and manipulated in the MRR region of DAT-Cre mice.

**Figure 3:**
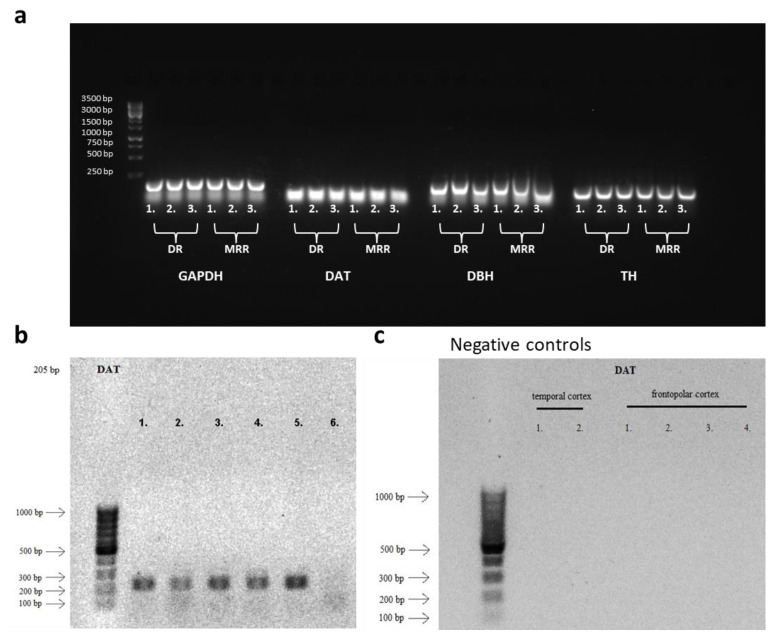
Amplification products obtained by RT-PCR. (**a**) In the wild-type mouse (C57BL/6J, the background strain for the Cre mice used in this study), both the dorsal raphe (DR) and median raphe region (MRR) samples showed measurable mRNA expression of dopamine transporter (DAT) and tyrosine hydroxylase (TH). Interestingly, as suggested in the literature, the noradrenergic marker dopamine-β-hydroxylase (DBH) was also detectable. Glyceraldehyde 3-phosphate dehydrogenase (GAPDH) was used as a housekeeping gene. (**b**) Samples of control human pontine raphe nuclei (which contain the human equivalent of mouse MRR) were obtained from the Human Brain Bank (Semmelweis University, Budapest, Hungary) and ran in gel electrophoresis for detection of DAT expression. The product displayed matched the length of DAT. (**c**) Negative controls from 6 cortical samples (2 from temporal cortex and 4 from frontopolar cortex). No DAT expression was observed in these two cortical areas.

Additionally, we were able to confirm the presence of DAT-positive cells both in the mouse and human brainstem, containing the MRR, by RT-PCR. In the mouse, not only DAT and TH, but also DBH mRNA expressions were found indicating that not only dopaminergic, but also noradrenergic neurotransmission is present (Figure 3a). Not only the MRR, but also the DR contained these markers. Moreover, in humans, the MRR-equivalent pontine raphe nucleus of the brainstem also expressed DAT (Figure 3b). However, the same expression was not detectable in cortical areas (Figure 3c).

### 2.2. Locomotion

**Figure 4:**
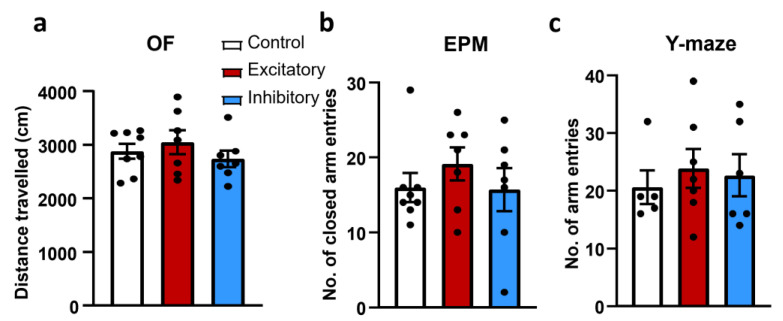
Locomotion after chemogenetic manipulation of median raphe region dopaminergic cells (DAT-MRR) in male dopamine transporter Cre mice. No difference was observed between treatment groups. (**a**) Distance travelled in cm in the Open Field test (OF). (**b**) Number of closed arm entries during the 5 min elevated plus maze (EPM) test. (**c**) Number of entries in the arms of the Y-maze. Data (*n* = 7–8) are expressed as average ± SEM. Dots represent individual values.

In order to see if general motility is affected by the dopaminergic neurons in the MRR, we investigated the mice’s locomotion during several tests, namely OF, EPM and the Y-maze. To manipulate neuronal cell activity, in the following experiments Cre-dependent DREADDs were expressed in the MRR of DAT-Cre mice with the help of adeno-associated viral vectors (AAV). Based on the expressed DREADDs, three groups were compared: Control (only RFP in the AAV), Excitatory (G_s_—pathway DREADD) or Inhibitory (G_i_—pathway DREADD). In DAT-Cre mice, manipulation of the DAT-MRR cells had no effect on locomotion in either studied parameters and tests (distance travelled in the OF, closed arm entries in EPM, total arm entries in Y-maze) (Figure 4).

### 2.3. Social Behavior

As the MRR is also implicated in social behavior [14,15,16,17], we used an extensive behavioral test battery to analyse the social behavior of the mice after exciting or inhibiting the dopaminergic neuronal activity of the MRR. During the habituation phase of the sociability test (introduction of the animals to two empty boxes) there was a side preference in frequency (F(1,19) = 5.439, *p* = 0.030) but not in duration of sniffing (F(1,19) = 0.014, *p* = 0.906) and none of the treatment influenced the time spent with the objects (F(2,19) = 1.282, *p* = 0.300).

**Figure 5:**
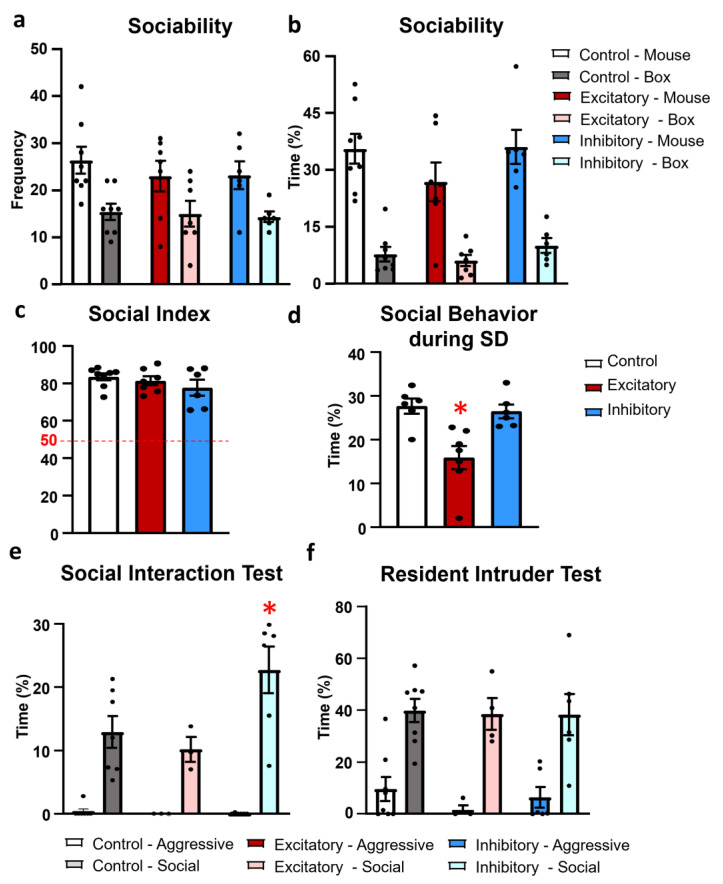
Results of social behavior tests. (**a**) Frequency of the interaction (sniffing) between the test mice and the empty cage (box) or caged mice (mouse). (**b**) Time of this interaction. (**c**) Social Index displayed the social preference toward the stimulus mice. For more details, see methods section. All groups performed above the chance level 50. (**d**) Time spent interacting with mice (caged familiar and caged unfamiliar mice) during social discrimination. Chemogenetic stimulation of the dopaminergic cells of the median raphe region (DAT-MRR) decreased social interest. (**e**) Time spent interacting with conspecific animal during social interaction test. Chemogenetic inhibition of DAT-MRR cells increased social interest. (**f**) Time spent interacting with conspecific animals during resident intruder test. Data (*n* = 6–8) are expressed as average ± SEM. Dots represent individual values. * *p* < 0.01 vs. control.

The introduction a juvenile mouse into one of the small cages significantly increased the frequency (F(1,18) = 35.155, *p* < 0.01) and time spent sniffing this cage (F(1,18) = 121.237, *p* < 0.01) (Figure 5a,b). However, the treatment had no effect on these parameters alone (frequency: F(2,18) = 0.287, *p* = 0.753; duration: F(2,18) = 1.458, *p* = 0.258), and did not interact with the presence of the stimulus mouse (frequency: F(2,18) = 0.356, *p* = 0.705; duration: F(2,18) = 0.925, *p* = 0.414). The index of social interest was above the 50% chance level in all the studied groups, suggesting a social preference (single sample *t*-test: Control: t(8) = 18.006, *p* < 0.01; Excitatory: t(7) = 13.114, *p* < 0.01; Inhibitory: t(6) = 6.492, *p* < 0.01) (Figure 5c).

During the social discrimination test (SD; 24 h after the CNO injection) an altered social interest (time spend sniffing both unfamiliar and familiar mice) was detectable (F(2,16) = 10.522, *p* < 0.01). More precisely, during this phase the Excitatory group dealt with the stimulus animals significantly less than the Control (*p* < 0.01) or Inhibitory groups (*p* < 0.01) (Figure 5d).

During the 10 min of the anxiogenic social interaction test (SIT) the animals initiated more friendly than aggressive encounters with each other (frequency: F(1,13) = 414.678, *p* < 0.01), without any influence of the treatment (F(2,13) = 1.423, *p* = 0.276). The main parameter, the time spent sniffing each other showed not only a significantly more time spending with friendly than aggressive behavior (F(1,13) = 61.476, *p* < 0.01), but also a significant interaction between the treatment and social behaviors (F(2,13) = 4.410, *p* = 0.034) (Figure 5e). The post hoc comparison revealed that the Inhibitory group spent significantly more time exhibiting friendly social behavior than the Control (*p* < 0.01) or Excitatory groups (*p* < 0.01) without significant differences in aggressive behavior.

During the 10 min of the resident–intruder test (RIT) the animals initiated more contact (F(1,15) = 190.106, *p* < 0.01) and spent significantly more time carrying out friendly than aggressive encounters (F(1,15) = 48.138, *p* < 0.01) (Figure 5f). In this case, the treatment had no effect on social (frequency: F(2,15) = 1.134, *p* = 0.347; duration: F(2,15) = 0.020, *p* = 0.979) or on aggressive behavior (frequency: F(2,15) = 1.042, *p* = 0.376; duration: F(2,15) = 0.727, *p* = 0.499).

### 2.4. Anxiety

**Figure 6:**
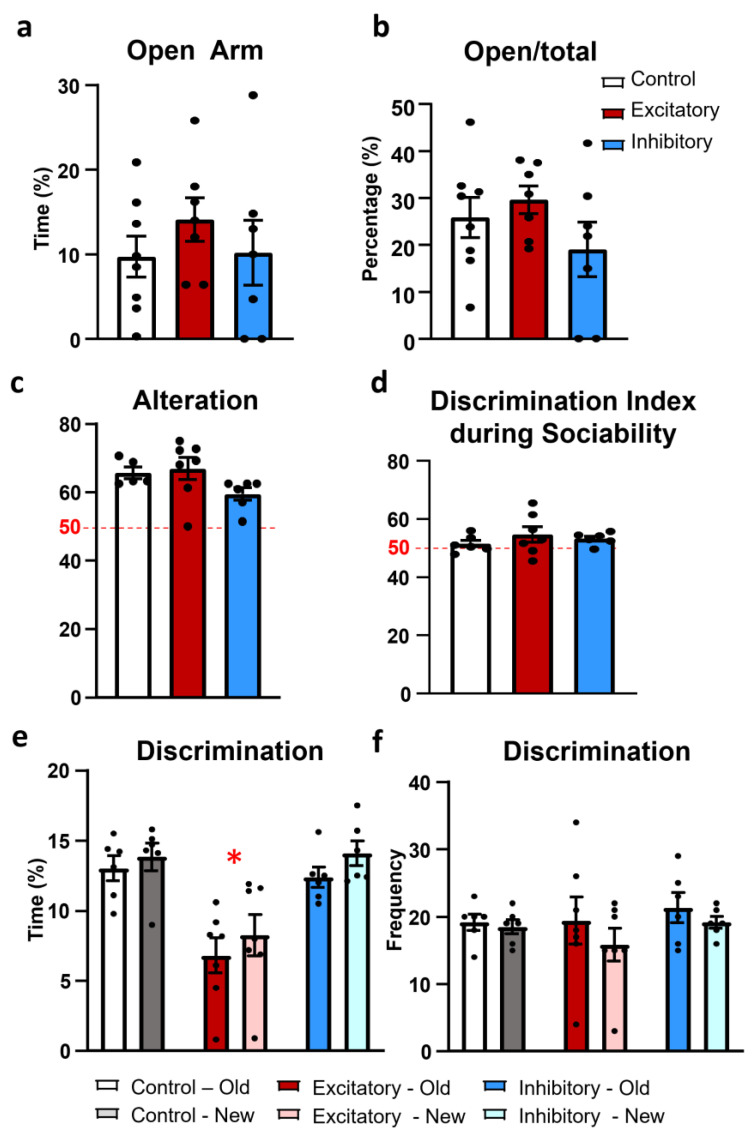
Results of anxiety and memory tests. (**a**) The time spent in the open arm in the elevated plus maze during the 5 min observation was without group difference. (**b**) The open/total arm entries shows the index of anxiety [50] without group difference. (**c**) Percentage of “good” alternations in the Y-maze (see Section 4.3.6). All animals performed above the chance level (marked red, 50). (**d**) Discrimination index during the social discrimination test. Animals were not able to distinguish between familiar and unfamiliar mice 24 h after “sampling” (no significant difference from the 50% chance level). (**e**) The time the test animal spent investigating old and new stimulus animal during the social discrimination test. Chemogenetic stimulation of the dopaminergic cells of the median raphe region (DAT-MRR) decreased overall social interest. (**f**) Frequency with which the animals spent with the caged conspecific animals during the social discrimination test revealed no difference between groups. Data (*n* = 6–8) are expressed as average ± SEM. Dots represent individual values. * *p* < 0.01 vs. control and inhibitory groups.

As anxiety may influence social behavior, especially during the social interaction test, we analyzed this parameter separately, in the elevated plus maze (EPM) test. There was no difference between the treatment groups in the time spent in the open arm (F(2,19) = 0.654, *p* = 0.530) (Figure 6a) or in the locomotion-independent measure of anxiety: the open arm entries (F(2,19) = 1.371, *p* = 0.277) (Figure 6b).

### 2.5. Memory

The MRR extensively innervates the hippocampus [7,8], a known center of learning and memory, and the prefrontal cortex, which is implicated in short-term working memory [51,52]. Thus, we tested whether the dopaminergic neurons in this area might be responsible—at least partly—for the memory-influencing effect of the MRR. All animals showed intact short-term memory exceeding the 50% chance level in the Y-maze test (Control: t(5) = 9.313, *p* < 0.01; Excitatory: t(7) = 5.161, *p* < 0.01; Inhibitory: t(6) = 5.208, *p* < 0.01) (Figure 6c). However, there was no significant difference between the groups (F(2,15) = 2.408, *p* = 0.123).

One day after the sociability test, the social recognition of a previously known mouse did not show statistical significance (F(2,16) = 0.756, *p* = 0.485) (Figure 6d). However, DAT-MRR stimulated animals spent less time investigating the stimulus animals (F(2,16) = 10.522, *p* = 0.001) compared to the Control and Inhibitory groups (*p* = 0.001; Figure 6e), with similar sniffing frequencies throughout the groups (F(2,16) = 0.429, *p* = 658; Figure 6f). Note that there was no direct manipulation of the DAT-MRR cells right before this test.

## 3. Discussion

Our results suggest that the MRR contains dopaminergic neurons (both in mice and in humans) and that these cells do not influence locomotion, anxiety or memory; however, their stimulation in mice decreased social behavior during the social discrimination test, whereas their inhibition increased the friendly social behavior in the social interaction test.

The presence of DAT in the human pontine raphe nucleus confirmed the translational value of our results. Despite the adequate RNA content (see Supplementary Table S1), only a very faint expression was observed in sample 6. It is possible that this person was in agony for a longer time than the others (see Supplementary Table S2) and his oxygen supply may have been permanently reduced due to previous pneumonia as well, which may negatively affect RNA stability [53]. However, it cannot be ruled out that the sampling does not always succeed with the same accuracy, which is also why there may be differences in the DAT expression of the samples. The absence of DAT mRNA in cortical areas was in line with a previous rat study [52] and can be explained by its expression in the cell bodies rather than on axon terminals.

Table 1 summarizes all of the changes observed during behavioral experiments performed on DAT-Cre mice in this study.

In the tests used to measure the locomotor activity of the mice (OF, closed arm entries in EPM and total arm entries in Y-maze) no difference was found between the Excitatory, Inhibitory and Control groups. According to the literature [54,55,56], DA is directly responsible for the locomotor activity. However, it was mainly the nigrostriatal pathway that was implicated in this behavior. Additionally, a previous study has shown that injecting different drugs (GABA agonists, opioid agonists) into the MRR may lead to hyperlocomotion [57]. Not all effect was antagonized by haloperidol (D2 antagonist) injection, suggesting a DA-dependent as well as an independent MRR-related influence on locomotion [57,58]. We have to add, due to its vast projection [59], it is not easy to determine which pathway the DAT-MRR is involved in. Nevertheless, in light of the current findings, we might conclude that dopaminergic cells from the MRR might have a different role than the nigrostriatal pathway.

During the habituation phase of the sociability test, there was no sign of fear of the object in any group tested. Although DA is implicated in fear, it is more connected to the extinction than the realization of the fear response [60,61] and the ventral tegmental area (VTA) has been suggested as the main source [60]. However, D1 receptors in the prefrontal cortex have a role in the acquisition of contextual fear conditioning [62]. We cannot entirely rule out that the dopaminergic cells of the MRR may project into this area, as DA may co-localize with VGluT3 [63], and previous studies showed VGluT3 innervation from the MRR to the prefrontal region [64]. Additionally, our recent study suggested that MRR VGluT2 neurons regulate the acquisition of negative experience in mice [22]. However, it is still not yet known if there is a DA-VGluT2 interaction in the MRR. Further studies are required to address the detailed role of the DA cells in the MRR in the context of fear.

In the second phase of the sociability test, social interest was investigated, but no changes were observed among the groups. All animals displayed more interest toward the conspecific stimulus mice rather than the empty cage. In contrast to our results, Bariselli et al. [65] described that the inhibition of DA neurons decreased the sociability among conspecifics; however, they investigated the DA neurons of the VTA. In our experiments, during the social discrimination phase, excitation of the DAT-MRR cells resulted in a decrease in social interest toward both conspecific stimulus mice. This result corroborates the findings of the social interaction test, where the inhibition of the DAT-MRR cells increased the friendly social behavior. In contrast, Liu et al. [66] found that the dendritic cell fact 1 (Dcf1) knockout mice displayed social interaction deficit and it could have been reverted by DA or a D1 receptor agonist, suggesting that a lower rather than an enhanced dopaminergic tone will lead to reduced social interest. This difference might be due to the divergent role of DA on different brain areas and also the use of different genetic techniques. Nevertheless, our results suggest that the dopaminergic neurons in the MRR might be also involved in the regulation of social behavior, an important manifestation of normal as well as pathological behavior.

In the resident–intruder test, no statistically significant difference was observed between the studied groups. This might be due to the fact that social behavior and ethological aggression are regulated by slightly different mechanisms [67]. Another possible explanation is that the later experiment was carried out in the dark, thus, was less distressing for the nocturnal animals. Indeed, previously it was shown that the outcome of the test is highly dependent on the anxiogenic nature of the environment. Although both tests (SIT and RIT) represent a social challenge and induce similar behaviors, they involve different contexts, and thereby different levels of anxiety. For example, Haller et al. [68] showed that the cannabinoid receptor 1 (CB1) KO mice were more aggressive during RIT, while they were less aggressive during SIT compared to the wild-type mice, which was explained by the different stressfulness of the tests (e.g., familiar vs. non-familiar environment; light vs. dark during the test).

Although we were not able to find any anxiety-related effect in the EPM test, this test was also performed in the dark, where the CB1 KO animals also behaved normally [68]. However, our EPM results were in contrast with the findings of Bahi and Dreyer [69], presenting a decreased anxiety both in the OF and EPM after silencing the DAT in the nucleus accumbens. Once again, this variation is probably due to the different brain region targeted, and the environmental conditions may also have influenced the outcome (the mentioned study was carried out during the animals’ light, inactive phase). Nonetheless, given the successful history of evaluating the anxiety-like behavior in mice with the EPM test [70,71,72], our results might indicate that the manipulation of DAT-MRR cells does not have a strong effect on anxiety control.

Previous studies have shown that the acquisition and consolidation of memory involve dopaminergic activity [73,74]. In addition, a recent study found that a decrease in dopaminergic cells might be a neurocognitive signature of Alzheimer’s disease [75]. All these studies suggest a close relationship between DA and memory. We assessed working memory using the Y-Maze and social memory using SD without treatment effects. In the SD test, the test mice acted as if they had never met the familiar mice. This result corroborates the notion that social memory only lasts for a few hours under laboratory conditions [76,77]; although it has been reported that vasopressin release as well as group housing could prolong this effect [77,78,79]. Our results might indicate that DAT-MRR is not involved in either working or social memory. However, it might have a role in spatial memory. Indeed, the injection of a D1/D5 antagonist into the CA1 region of the hippocampus decreased the SD abilities at 24 h [80]. As VGluT3-MRR cells projecting to the hippocampus [64] may be co-localized with DA, they might provide the source of DA in the hippocampus. Although, these assumptions need further confirmation.

Our experiments have certain limitations that need to be addressed in future studies. (i) We tried to use the most optimal tests to measure each behavior, but in order to have a general view, we used a long test duration and repeated the CNO injections. However, our previous optogenetic manipulation showed that a single 5 min stimulation of the MRR may induce long lasting, plastic changes in the animal’s behavior [81]. (ii) Although one might expect that the Excitatory and Inhibitory groups would behave in opposite ways, our results did not confirm this assumption. Nevertheless, it this not that surprising as the two types of DREADD sequences affect different signaling pathways (Gq and Gi). Moreover, stimulation seems to be a more active process, while inhibition mostly diminishes the effect of other stimulatory signals.

Our results further extend our understanding on the role of dopaminergic cells in our brain and raise some questions that could be addressed in future research. This is of utmost importance as many people worldwide take drugs influencing their dopaminergic system [82] such as L-DOPA for treatment of Parkinson’s disease, [83], Aripiprazole for the treatment of schizophrenia and bipolar disorder [84,85], Tetrabenazine for the treatment of Huntington’s Disease [86] and Pramipexole for the treatment of Restless legs syndrome [87] and essential tremors [88], which might have unwanted behavioral side effects leading to discontinuation of the pharmacotherapy. We focused on social behavior as it is a fundamental property of every-day interactions and serves as the basis for survival and reproduction [89]. Understanding the mechanisms behind this behavior could help scientists to provide a better treatment for those who suffer from psychological and psychiatric disorders.

## 4. Materials and Methods

### 4.1. Animals

Adult male mice of C57BL/6J background containing the Cre enzyme under the DAT [90] promoter were obtained from The Jackson Laboratory (Bar Harbor, ME, USA; Stock No.: 006660), and local colonies were maintained at the Institute of Experimental Medicine, Budapest, Hungary. The experimental animals were all heterozygous for the Cre enzyme (see reduced DAT expression in homozygous mice [91]) and were from C57BL/6J mothers to avoid any maternal impact. The mice were group housed (2–3 mice/cage) until the beginning of the behavioral examination, when individual housing began to enhance social interest. Male juvenile (30–45-day-old) C57BL/6J mice were used as stimulus animals for the sociability and resident intruder tests as previously suggested [92,93]. For RT-PCR, C57BL/6J adult male mice from the same local colony were used. The animals were kept in standard environment (21 ± 1 °C, 12 h light/dark cycle with light on at 7 p.m.) and had access to food (standard laboratory chow; Charles River, Hungary) and water ad libitum. The behavioral examinations were at the beginning of the dark, active phase under red light, as it is known that rodents are nocturnal animals [94].

The experiments were approved by the Workplace Animal Welfare Committee of Institute of Experimental Medicine and National Scientific Ethical Committee on Animal Experimentation of Hungary (PEI/001/33-4/2013, PE/EA/254-7/2019) and were performed according to the European Communities Council Directive recommendations for the care and use of laboratory animals (2010/63/EU).

### 4.2. Surgery

The mice were anesthetized with intraperitoneal (i.p.) injection of 0.2 mL anesthetics (0.5 mL of ketamine, 0.1 mL of xylazine and 2.4 mL of physiological saline) (Medicus Partner, Biatorbágy, Hungary). At the beginning of the operation, as well as during the next two days, buprenorphine (Bupaq (Gedeon Richter Plc., Budapest, Hungary); 0.1 mg/kg) was given to them subcutaneously as a painkiller.

Three different virus constructs were used containing the fluorophore mCherry (referred later as red fluorescent protein, RFP) and/or DREADD sequence between two loxP loci and under the control of a neuron-specific Syn promoter. In DAT-Cre mice these vectors limit the expression of RFP and DREADD to dopaminergic cells (Figure 2) [90]. The rAAV8/hSyn-DIO-hM3D(Gq)-mCherry (5.9 × 10^12^ gc/mL, Addgene, Watertown, MA, #44361) construct activates, while the rAAV8/hSyn-DIO-HM4D(Gi)-mCherry (1.9 × 10^13^ gc/mL, Addgene, Watertown, MA, #44362) construct inhibits, the cells. Controls were injected with rAAV8/hSyn-DIO-mCherry (4.1 × 10^12^ gc/mL, Addgene, Watertown, MA, #50459) virus vector. Twenty nanoliters of the virus were injected via glass pipettes (tip diameter 20–30 μm) connected to a MicroSyringe Pump Controller (World Precision Instruments, Sarasota, FL, USA) into the MRR of mice fixed in a stereotaxic system (David Kopf Instruments, Tujunga, CA, USA). The coordinates were the following: AP: −4.1 mm of the bregma; DV: 4.6 mm; and 0 mm lateral to midline.

### 4.3. Behavioral Analysis

Tests were carried out between 9–13 h in a separate room of the animal facility and were recorded by digital camera (Samsung SNB 7000). The order of tests was the following: sociability, social interaction (SIT), resident intruder (RIT), elevated plus maze (EPM) and y-maze (Figure 1). In order to activate the DREADD, CNO (Tocris, Budapest, Hungary; 1 mg/kg dissolved in 10 mL saline, injected in 0.1 mL/10 g volume) was injected intraperitoneally 30 min before each behavioral test. To balance out any possible CNO-induced effects all the groups (including controls) received CNO. Data were analyzed later by computer-based event recorder H77, Noldus EthoVision (15.0; Wageningen, The Netherlands) or Solomon Coder (beta 19.08.02; Budapest, Hungary) by an experimenter blind to the treatment groups. Each test apparatus was cleaned with 20% ethanol, and dried prior to the next animal being introduced.

#### 4.3.1. Open Field Test (OF)

The animals were placed in a non-transparent white plastic box (40 cm × 36 cm × 19 cm) for 5 min under infrared light. EthoVision software was used to measure the distance traveled and the time spent in the peripheral and central zones (see [95], for further details about the protocol). The locomotor activity and anxiety-like behavior were measured.

#### 4.3.2. Sociability Test

The sociability test directly followed the open field test without further CNO injection. It consisted of three phases: habituation, sociability, and social discrimination. In the habituation phase, two small cages were put inside the plastic box, placed on opposite sides and the test animal had 5 min to explore and become familiar with the cages. During the sociability phase one stimulus mouse (juvenile male C57Bl6 mice [16,96]) was put inside one of the cages. The test mice then had another 5 min to explore the test cage. Next, social discrimination was performed 24 h after the sociability phase without further CNO injection. During this phase we put the same stimulus mice on the opposite edge of the cage (in order to avoid side preference), and we put a new stimulus mouse under the other cage. Here a comparison was made between the time that the test mice spent with the new and old stimuli mouse. This phase also lasted 5 min. This test examined the lasting memory effect of the stimulation/inhibition of MRR.

The time and frequency of sniffing the small cages (either empty or containing a conspecific, familiar or unfamiliar) were measured by H77 event recorder.

The sociability index (SI) and social discrimination (SD) were calculated using Equation (1):

(1) $$SI=\frac{t_{mouse}}{t_{mouse}+t_{cage}}\times 100\;\;\;\;SD=\frac{t_{new}}{t_{new}+t_{old}}\times 100$$

where t_mouse_: time spent sniffing the box containing a juvenile mouse; t_cage_: time spent sniffing the empty cage; (SD): social discrimination; t_new_: time spent with a new mouse; t_old_: time spent with the old, familiar mouse.

#### 4.3.3. Social Interaction Test (SIT)

The test took place in two phases, habituation and social interaction [97]. The first phase happened on the day before the social interaction phase, where the mice were put alone in a test box (new, unknow environment) for 10 min without CNO injection. The same box was used on the next day for the second phase. The habituation phase was carried out to make the animals familiar with the new environment, and to enhance social interaction. Both the unfamiliarity of the environment and the lighting during the test enhance anxiety, thereby the results of this test might be also interpreted in relation to anxiety [97].

On the second day, two test mice (same treatment) were put together for 10 min, 30 min after an intraperitoneal injection of CNO (1 mg/10 mL/kg). During these 10 min, the animal’s behavior was recorded. The following parameters were taken into consideration: aggressive, defensive, social and other. The aggressive behavior included any type of aggressive and abnormal behavior from the mice, e.g., biting, chasing, mating. Defensive behavior was categorized as whenever the mouse was fleeing from attacks, and when they were hitting back after being attacked. The animal behaviors were labelled as social whenever one animal was sniffing the other. The “other” criteria were used when the animals were not interacting with each other. The time and frequency of these behaviors were analyzed by Solomon Coder program.

#### 4.3.4. Resident Intruder Test (RIT)

For this experiment, the test mice (resident) were isolated in a cage for a week starting on day 1 of testing, during the CNO injection. During this period the cages were not cleaned as olfactory cues help to determine the territoriality of the animal [98].

The test started when one stimulus mouse (intruder) was introduced to the cage of the test mice. For this experiment, we prioritized younger and smaller stimulus mice, as they tend to be submissive to older/bigger mice. Our intent with this was to observe only the aggression coming from the dominance of the test mice. The test was 10 min, and the behavior was classified in the same way as it was during the SIT using the Solomon Coder program.

#### 4.3.5. Elevated plus Maze Test (EPM)

The test is based on the natural explorative behavior of the rodents in new environments and is widely used to assess anxiety in laboratory animals [99]. Due to the natural aversion of the mice to elevated and open places, they are expected to spend more time in the closed arm [100].

The EPM apparatus consisted of two open arms and two closed arms (with walls and open at the top; 67 cm × 7 cm × 30 cm) crossed in the middle perpendicularly with each other. We put the mice in the centrum of the EPM and let them freely explore the apparatus for 5 min. The number of entries into the arms and the time spent in them were measured with a H77 event recorder (Budapest, Hungary). The number of closed arm entries reflects locomotion, while time spent in the open arms is the widely accepted measure of anxiety. The number of entries into the open/(open + closed) arm × 100 is considered as a locomotor-independent measure of anxiety [17,50,101,102,103].

#### 4.3.6. Y-Maze Test

This test detects the ability of the short-term learning and memory of the rodent. The apparatus consists of three arms (A, B and C) at 120 degrees, connected by a central zone (CZ). In this experiment, a test mouse was put into an arm, and while it explored the maze, it was expected to enter the other arms consecutively. An animal with a good short memory is able to remember the arms where it has been just before and will demonstrate a propensity to visit the less recently visited arm (e.g., “good” alteration: ABC, BCA, CAB, ACB, CBA, BAC). On the other hand, a mouse with poor short memory, will keep entering the last visited arm [104].

The animals were tested for 5 min and the order of visited arms were observed on the video recordings. In order to calculate the percent (%) alternation the formula below was used (Equation (2)).

(2) $$\%Alt=\frac{n_{”good”alterations}}{n_{armentries}-2}\times 100$$

where (% Alt): percent alternation; n_”good” alterations_: number of “good” alterations; n_arm entries_: total number of arm entries.

### 4.4. Immunohistochemistry

Upon termination of the experiments (i.e., 6 weeks after AAV injection) mice were deeply anesthetized and transcardially perfused with 0.1 M phosphate-buffered saline (PBS) for 1 min, and then with 4% (*w*/*v*) paraformaldehyde in PBS for 20 min. Brains were taken out, and post-fixed for 24 h in fixative at +4 °C before being cryoprotected with 20% glucose-PBS solution for 24 h at +4 °C. Thirty μm thick coronal sections were prepared on a sliding microtome.

#### 4.4.1. Confirmation of Dopaminergic Cells in the MRR

On selected brain slices of control-virus-injected mice, double fluorescent immunohistochemistry was conducted. DA was visualized by positive TH and negative DBH immunohistochemistry together with the RFP immunohistochemistry visualizing the dopaminergic cells in DAT-Cre mice after control virus injection.

Primary antibodies were diluted in PBS (a-TH (host: rabbit), 1:1000, DiaSorin, Stillwater, MN, USA, or a-DBH (host: rabbit), 1:1000, PA1-18314, Invitrogen, Carlsbad, CA, USA) and brain samples were incubated for 3 nights. After washing, sections were incubated in secondary antibody solution for 1 h (Biotin SP conj. goat a-rabbit 1:1000, Vector, CatNo: BA 1000; diluted in PBS). Slices were incubated in Extravidin-peroxidase (Lot#053M4811V, Sigma-Aldrich, Budapest, Hungary) for 1 h. After multiple washes, Fictiramin (488 nm) fluorophore was used for 10 min. Then, anti-RFP primary antibodies (incubation time: 2 nights) and Alexa fluor 488 fluorescent secondary antibodies (incubation time: 2 h) were used for DREADD immunolabeling. Sections were mounted with Mowiol.

In a separate set of animals 2 weeks after control virus injection, TH and DBH co-positivity were studied on the same slides together with RFP expression in a triple fluorescent immunohistochemistry. On parallel sections, RFP and DAT co-expression was also studied. For these stainings, the following antibodies were used: Primaries: 1:100,000 a-RFP, sheep, acquired from Csaba Fekete at the Institute of Experimental Medicine, Budapest, Hungary; 1:1000 a-DAT, rat, MAB369, Sigma-Aldrich, Budapest, Hungary; 1:300 a-TH, mouse, #45648, Cell Signalling Technology, Danvers, MA, USA; 1:500 a-mouse Fab fragment, donkey, 715-007-003, Jackson ImmunoResearch, West Grove, PA, USA; 1:2000 a-DBH, rabbit, EPR20385, Abcam, Cambridge, UK. Secondaries: 1:500 a-sheep A594, donkey, A-11016, Invitrogen, Carlsbad, CA, USA; 1:500 a-rat A488, donkey, A-21208, Invitrogen, Carlsbad, CA, USA; 1:1000 biotin conjugated a-mouse, donkey, 715-065-150, Jackson ImmunoResearch, West Grove, PA, USA; 1:500 a-rabbit A488, A-21206, Invitrogen, Carlsbad, CA, USA; and 1:500 streptavidin conjugated Pacific Blue fluorescent dye, S11222, Thermo Fisher Scientific, Budapest, Hungary. Slices were mounted in PBS and covered with glycerol.

Sections were evaluated with a fluorescent C2 confocal laser-scanning microscope (Nikon Europe, Budapest, Hungary, 100× and 20× objective).

#### 4.4.2. Verification of the Virus Injection

The RFP signal was enhanced by a nickel-3,3′-diaminobenzidine (Ni-DAB) immunohistochemistry carried out with a rabbit anti-RFP primary antibody and a biotinylated secondary anti-rabbit antibody coupled to an avidin–biotin complex (ABC). The RFP was labeled with a rabbit polyclonal antibody. The primary antibody (1:4000) was detected by biotinylated anti-rabbit goat serum (1:1000) and avidin–biotin complex diluted in Tris buffer (1:1000, Vectastain ABC Kit, Vector Laboratories, Newark, CA, USA). The peroxidase reaction was developed in the presence of diaminobenzidine tetrahydrochloride (0.2 mg/mL), nickel–ammonium sulphate (0.1%) and hydrogen peroxide (0.003%) dissolved in Tris buffer. The sections were mounted on glass slides and covered with a DPX mounting medium. The virus-infected area was localized on micrographs by using an overlay of the stereotaxic atlas images on the series of images of the MRR [49].

**Figure 7:**
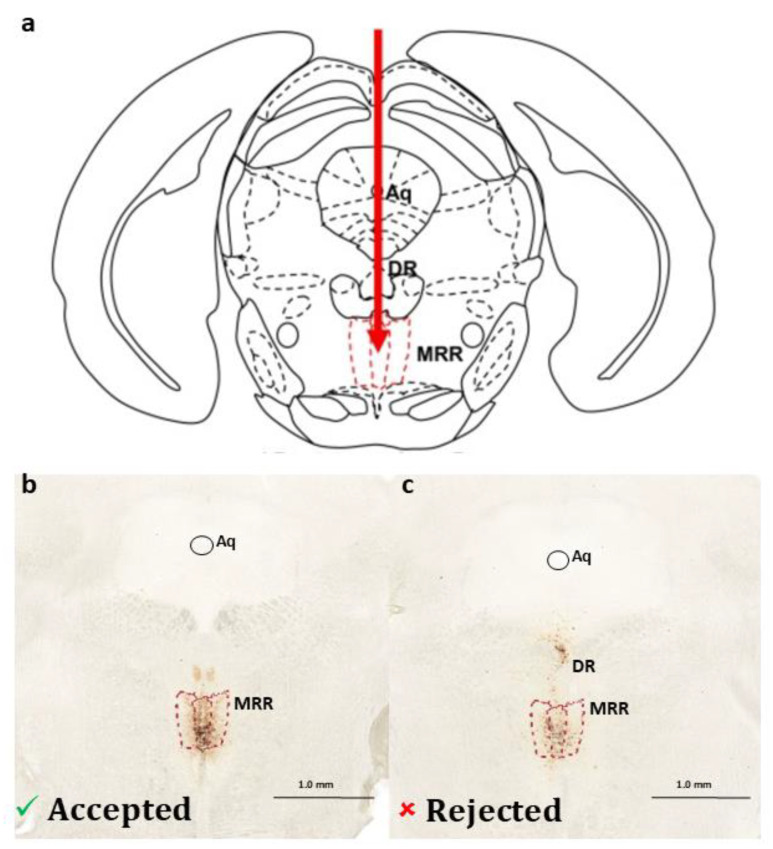
Virus injection details. (**a**) Schematic illustration of the targeted mouse median raphe region (MRR) in coronal section according to the Mouse Brain in Stereotaxic Coordinates [49]. (**b**) Representative image of an appropriate virus expression (visualized by nickel-3,3′-diaminobenzidine staining for the reporter protein red fluorescent protein [RFP]) in the MRR of a dopamine-transporter-Cre mice 6 weeks after 10 nL rAAV8/hSyn-DIO-HM4D(Gi)-mCherry (Addgene, Watertown, MA, #44362) injection. (**c**) Representative figure of a rejected virus expression with staining both in the dorsal raphe (DR) and MRR. Aq: cerebral aqueduct. Coordinates: anterior–posterior: −4.1 mm; lateral: 0.0 mm; dorso-ventral: −4.6 mm from Bregma.

If there was no staining, or it was unilateral, or other brain regions (e.g., DR) were also stained, the animal and the data belonging to it were excluded from the statistical analysis (Figure 7).

### 4.5. Verification of the Presence of DA in the Raphe by RT-PCR

For measurements in mice, wild-type C57BL6/J animals were sacrificed, and their DR and MRR region were dissected by punch needles, before being fresh frozen on dry ice. Samples were kept on −80 °C until further preparations for one-step PCR.

In the case of human samples, the study was approved by the Hungarian Medical Research Council—Scientific and Research Ethical Committee (Egészségügyi Tudományos Tanács—Tudományos és Kutatásetikai Bizottság, #40197-2/2019/EKU), in accordance with the Ethical Rules for Using Human Tissues for Medical Research in Hungary (HM 34/1999) and the Code of Ethics of the World Medical Association (Declaration of Helsinki). Post-mortem brain samples were obtained from the Human Brain Tissue Bank, Semmelweis University (Budapest, Hungary). The brains of twelve individuals who died of natural causes were used in the measurement. The details of the samples can be found in Supplementary Tables S1 and S2.

#### 4.5.1. RNA Extraction and cDNA Synthesis

For mouse samples, frozen samples were thawed and total RNA was processed according to RNeasy^®^ Mini Kit instructions (QIAGEN, Germantown, MD, USA, #74106). Reverse transcription (High-Capacity RNA-to-cDNA™ Kit, Thermo Fisher Scientific, Budapest, Hungary #4387406) and one-step PCR (DreamTaqTM Green PCR Master Mix, Thermo Fisher Scientific, Budapest, Hungary, K1085) were performed by Biometra Tone (Analytik Jena, Jena, Germany). The following primers were designed using Primer-BLAST (NCBI) and were acquired from Integrated DNA Technologies (IDT; Coralwille, Iowa, USA): glyceraldehyde 3-phosphate dehydrogenase (GAPDH, housekeeping gene), DAT, TH and DBH (see Supplementary Table S3).

For human samples, total RNA was isolated by using the RNeasy^®^ Mini Kit according to the manufacturer’s instructions. RNA was diluted into RNase-free water. The quality and quantity of extracted RNA were determined using NanoDrop ND-1000 Spectrophotometer (Thermo Fisher Scientific, Budapest, Hungary), and only those with A260/A280 ratio between 1.8 and 2.1 were used in subsequent experiments (Supplementary Table S1). The isolated RNA concentration was calculated and normalized with RNase-free water and reverse transcribed into cDNA using a SuperScript II reverse transcriptase kit (Invitrogen, Carlsbad, CA, USA) (see Supplementary Table S2 for a list of all samples.). After 10-fold dilution, 2.5 μL of the resulting cDNA was used as template in PCR. The PCR reactions were performed on CFX-96 C1000 Touch Real-Time System (Bio-Rad Laboratories, Hercules, CA, USA) with iTaq DNA polymerase (Bio-Rad Laboratories, Hercules, CA, USA) in total volumes of 12.5 μL under the following conditions: 95 °C for 3 min, followed by 35 cycles of 95 °C for 0.5 min, 60 °C for 0.5 min and 72 °C for 1 min. All the determinations were conducted in duplicate. The primers used for RT-PCR were synthesized by IDT (Coralville, IA, USA) and used at 300 nM final concentration. Sequences of primers are listed in Supplementary Table S3.

#### 4.5.2. Agarose Gel Electrophoresis

One third of the amplified PCR product was loaded in an agarose gel (1.2%) containing Eco Safe nucleic acid staining solution (1:20,000, Pacific Image Electronics, New Taipei City, Taiwan) and the electrophoresis was conducted in 1× TAE (40 mM Tris-Acetate, 1 mM EDTA, pH 8.0) buffer. The separation voltage applied was 100 V. After the electrophoresis the DNA bands were visualized by UV transillumination.

### 4.6. Statistics

Data were analyzed with StatSoft Statistica 13.0 (Tulsa, OK, USA) utilizing a single sample *t*-test (Sociability Index, spontaneous alteration, Discrimination Index, in comparison to 50%), one-way analysis of variance ANOVA (parameter: treatment) or repeated-measure ANOVA (Sociability, social interaction, resident-intruder and Social Discrimination) followed by Fisher LSD post hoc comparison. Data are expressed as mean ± SEM and *p* < 0.05 was considered statistically significant. In general, the behavioral data were expressed as % of investigated time period to correct for possible missing frames of the video recordings. All marks on figures represent the results of post hoc comparison, while the main ANOVA effects are described in the text.

## Figures and Tables

**Table 1:** Summary of results.

Function	Test	Excitatory	Inhibitory
Locomotion	OF	∅	∅
	EPM	∅	∅
	Y Maze	∅	∅
Social Behavior	Sociability	Decreased social interest	∅
	Social Interaction	∅	Increased social behavior
	Resident Intruder	∅	∅
Anxiety	EPM	∅	∅
Memory	Y Maze	∅	∅
	Social discrimination at 24 h	∅	∅

∅: no difference between Control and this group.

## Data Availability

Data are available upon request.

## Supplementary Materials

The following supporting information can be downloaded at: https://www.mdpi.com/article/10.3390/ijms25084315/s1.
