# Supplementary material for: The Dopaminergic Cells in the Median Raphe Region Regulate Social Behavior in Male Mice
Source: Int J Mol Sci. 2024 Apr 13;25(8):4315. doi: 10.3390/ijms25084315 (PMC11050709; doi:10.3390/ijms25084315)
Supplement: Supplementary file 1 [file ijms-25-04315-s001.zip › ijms-2948381-supplementary.pdf]

Supplementary Materials

Table S1. List of human brain samples and their respective RNA concentrations after isolation and RNA quantity after dilution.

Sample ID	Group	Area	RNA concentration in sample (ng/µL)	RNA quantity after dilution (ng)
**1**	Control	pontine raphe nucleus	206.60	1000
**2**	Control	pontine raphe nucleus	144.50	1000
**3**	Control	pontine raphe nucleus	590.20	1000
**4**	Control	pontine raphe nucleus	1048.20	1000
**5**	Control	pontine raphe nucleus	979.90	1000
**6**	Control	pontine raphe nucleus	225.30	1000
**1**	Control	temporal cortex	210.99	1000
**2**	Control	temporal cortex	457.82	1000
**1**	Control	frontopolar cortex	263.02	1000
**2**	Control	frontopolar cortex	263.31	1000
**3**	Control	frontopolar cortex	318.17	1000
**4**	Control	frontopolar cortex	232.36	1000

Table S2. Details of the human brain samples.

Brain #ID	Sample ID	Area	Gender	Age	Post-mortem delay (h)	Cause of death
**#186**	1	pontine raphe nucleus	female	56	5	myocardial infarction
**#209**	2	pontine raphe nucleus	male	52	4.5	myocardial infarction
**#211**	3	pontine raphe nucleus	female	56	6	cardiorespiratory insufficiency
**#216**	4	pontine raphe nucleus	male	53	5	pulmonary embolism
**#227**	5	pontine raphe nucleus	male	55	6	acute myocardial infarction
**#228**	6	pontine raphe nucleus	male	27	8	pneumonia
**#267**	1	temporal cortex	female	91	8	stroke
**#175**	2	temporal cortex	female	49	6	suicide (drug overdose)
**#211**	1	frontopolar cortex	female	56	6	cardiorespiratory insufficiency
**#267**	2	frontopolar cortex	female	91	8	stroke
**#159**	3	frontopolar cortex	male	48	6	suicide (hanging)
**#175**	4	frontopolar cortex	female	49	6	suicide (drug overdose)

Table S3. List of primers for PCR.

		Primer ID	Bases	Product Size (bp)
hDAT-E-1-F	20	GTC TGT TTG GAT TGA CGC GG	NM_001044.5	205
hDAT-E-1-R	20	ACT GTG CTT CTG TGC CAT GT		
mDAT-F	22	GGT GCT GAT TGC CTT CTC CAG T	NM_010020.3	112
mDAT-R	22	GAC AAC GAA GCC AGA GGA GAA G		
mDBH-F	22	GAG ACT GCC TTT GTG TTG ACC G	NM_138942.3	133
mDBH-R	22	CGA GCA CAG TAA CCA CCT TCC T		
mGAPDH-F	23	CAT CAC TGC CAC CCA GAA GAC TG	NM_001289726.2	153
mGAPDH-R	23	ATG CCA GTG AGC TTC CCG TTC AG		
mTH-F	23	TGC ACA CAG TAC ATC CGT CAT GC	NM_009377.2	107
mTH-R	22	GCA AAT GTG CGG TCA GCC AAC A		

A: adenine; C: cytosine; E: exon-exon junction; F: forward primer; G: guanine hDAT: human dopamine transporter; mDAT: mouse dopamine transporter; mDBH: mouse dopamine β-hydroxylase; mGAPDH: glyceraldehyde 3-phosphate dehydrogenase; mTH: mouse tyrosine hydroxylase; R: reversed primer; T: thymine.
